# Poly(Urethane-Acrylate) Aerogels via Radical Polymerization of Dendritic Urethane-Acrylate Monomers

**DOI:** 10.3390/ma11112249

**Published:** 2018-11-12

**Authors:** Maria Papastergiou, Aspasia Kanellou, Despoina Chriti, Grigorios Raptopoulos, Patrina Paraskevopoulou

**Affiliations:** Laboratory of Inorganic Chemistry, Department of Chemistry, National and Kapodistrian University of Athens, Panepistimiopolis Zografou, Athens 15771, Greece; mapapast@chem.uoa.gr (M.P.); aspasiakan@hotmail.com (A.K.); chritides@chem.uoa.gr (D.C.); grigorisrap@chem.uoa.gr (G.R.)

**Keywords:** acrylate, aerogel, dendritic, free radical polymerization, polymeric material, polyurethane aerogels, supercritical drying, porous networks

## Abstract

The purpose of this work was to investigate the effect of multifunctionality on material properties of synthetic polymer aerogels. For this purpose, we present the synthesis and characterization of monolithic dendritic-type urethane-acrylate monomers based on an aliphatic/flexible (Desmodur N3300), or an aromatic/rigid (Desmodur RE) triisocyanate core. The terminal acrylate groups (three at the tip of each of the three branches, nine in total) were polymerized with 2,2′-azobis(isobutyronitrile) (AIBN) via free radical chemistry. The resulting wet-gels were dried with supercritical fluid (SCF) CO_2_. Aerogels were characterized with ATR-FTIR and solid-state ^13^C NMR. The porous network was probed with N_2_-sorption and scanning electron microscopy (SEM). The thermal stability of aerogels was studied with thermogravimetric analysis (TGA). Most aerogels were macroporous materials (porosity > 80%), with high thermal stability (up to 300 °C). Aerogels were softer at low monomer concentrations and more rigid at higher concentrations. The material properties were compared with those of analogous aerogels bearing only one acrylate moiety at the tip of each branch and the same cores, and with those of analogous aerogels bearing norbornene instead of acrylate moieties. The nine-terminal acrylate-based monomers of this study caused rapid decrease of the solubility of the growing polymer and made possible aerogels with much smaller particles and much higher surface areas. For the first time, aliphatic/flexible triisocyanate-based materials could be made with similar properties in terms of particle size and surface areas to their aromatic/rigid analogues. Finally, it was found that with monomers with a high number of crosslinkable groups, material properties are determined by multifunctionality and thus aerogels based on 9-acrylate- and 9-norbornene-terminated monomers were similar. Materials with aromatic cores are carbonizable with satisfactory yields (20–30% *w*/*w*) to mostly microporous materials (BET surface areas: 640–740 m^2^ g^−1^; micropore surface areas: 360–430 m^2^ g^−1^).

## 1. Introduction

Aerogels are highly porous ultralight materials, consisting of low-density 3D assemblies of nanoparticles [1,2]. Regarding the origin of aerogels, it was Kistler who first prepared and then defined aerogels as “gels in which the liquid is replaced with a gas without collapsing the gel solid network” [3]. More recently, aerogels have been defined more broadly as “solid colloidal or polymeric networks of particles expanded throughout their entire volume by a gas” [4,5]. Aerogels are formed when wet-gels are dried by turning the solvent inside the pores into a supercritical fluid that is released like a gas. That procedure causes no substantial shape change, volume reduction, or network collapse [2].

Kistler’s work concentrated mainly on silica aerogels via an acid-catalyzed reaction with water glass, solvent exchange from water to ethanol, and supercritical fluid drying [3]. A few years later in 1942, Monsanto Corporation commercialized a product known as ‘aerogel’ under the trade name Santocel, according to Kistler’s procedure [6]. Because of attractive properties of silica aerogels (e.g., low values of thermal conductivity, very low density, high porosity, high surface area), those materials have found applications in space exploration [7,8], in nuclear reactors as Cerenkov radiation detectors [9,10,11], in catalysis [12,13], and in drug delivery [14,15]. Nowadays, several types of aerogels are known, including inorganic [16,17,18,19,20], organic (based on biopolymers [21,22,23,24] or synthetic polymers [25,26,27,28,29]), and hybrid inorganic/organic [30,31,32,33,34].

Among synthetic polymer aerogels, polyurethane (PU) aerogels have a prominent position, because they combine the versatility of the chemical composition of PUs with the properties of aerogels [35,36,37,38,39,40,41]. PU aerogels are synthesized from the reaction of polyisocyanates with polyols Equation (1) in the presence of a Lewis acid catalyst. As a result, the urethane group is the polymer repeat unit, but other functional groups (e.g., urea, ester, ether, aromatic groups) may also be present in the polymer structure depending on the monomer [42].

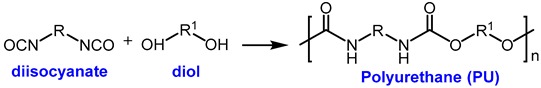
(1)

In addition, polymeric aerogels derived from the polymerization of star and dendritic monomers with urethane linkages have been reported; those include poly(urethane norbornene) [25,40,43] and poly(urethane acrylate) [40,43] aerogels. Those monomers consisted of an aromatic or an aliphatic core (coming from the triisocyanate) and three branches ending at one [40] or three [25] norbornene groups, or one [40] acrylate group. Comparing poly (urethane norbornene) materials, those with more peripheral norbornene groups (nine vs. three) had significantly improved properties, featuring higher crosslinking, larger porosities, and higher surface areas.

In this work, we expand our study to poly(urethane acrylate) aerogels obtained from dendritic monomers based on trifunctional core structures, bearing nine acrylate moieties. Those monomers were synthesized from the reaction of pentaerythritoltriacrylate (PETA) with an aliphatic/flexible triisocyanate (hexamethylene diisocyanate trimer, Desmodur N3300), or an aromatic/rigid one (Desmodur RE: triphenylmethane-4,4′,4″-triisocyanate, abbreviated as TIPM) using dibutyltindilaurate (DBTDL) as catalyst, and were polymerized using free radical chemistry initiated with 2,2′-azobis(isobutyronitrile) (AIBN). The resulting aerogels had a very high degree of crosslinking, high porosity, and high thermal stability. The properties of the new materials are compared with the properties of materials previously synthesized from similar trifunctional monomers bearing just one acrylate moiety at the tip of each branch (three in total) [40], and it was found that with the multifunctional monomers of this study, material properties such as density, particle size, and surface area all improved. Densities as low as 0.041 g cm^−3^, porosities as high as 97% *v*/*v*, particle sizes as low as 4.5 nm, and BET surface areas as high as 488 m^2^ g^−1^ are reported. For the first time to our knowledge, polyurethane aerogels from flexible/aliphatic triisocyanate cores were prepared with similar properties to analogous materials from rigid/aromatic triisocyanate cores.

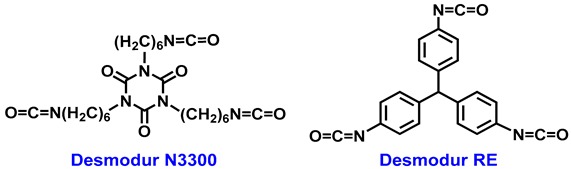


## 2. Materials and Methods

All procedures were carried out under an inert atmosphere, using Schlenk techniques on an inert gas/vacuum manifold or in a drybox (O_2_, H_2_O < 1 ppm) (MBRAUN, München, Germany). The inert gas used was Ar and it was passed through a BASF R-3-11 catalyst to remove traces of oxygen and moisture. All reagents and solvents were used as received. Desmodur N3300 (1,3,5-tris(6-isocyanatohexyl)-1,3,5-triazinane-2,4,6-trione) (in pure form) and Desmodur RE tris(4-isocyanatatophenyl)methane (TIPM; 27% *w*/*w* solution in ethyl acetate) were kindly donated by Covestro Deutschland GA (Leverkusen, Germany). Pentaerythritol triacrylate (PETA, SR444D) was kindly donated by Sartomer Arkema Group (Rieux, France). 2,2′-azobis(isobutyronitrile) (AIBN), dibutyltindilaurate (DBTDL), anhydrous acetone, and deuterated acetone (acetone-d^6^) were purchased from Acros Organics (Geel, Belgium).

Liquid ^1^H and ^13^C NMR spectra were obtained with a Bruker Avance DRX 500 MHz (Bruker, Billerica, MA, USA) (^1^H at 500.13 MHz and ^13^C at 125.77 MHz) in acetone-d^6^ at room temperature. ^13^C cross-polarization magic angle spinning (CPMAS) NMR spectra were obtained with a 600 MHz Varian spectrometer (Varian, Palo Alto, CA, USA) operating at 150.80 MHz for ^13^C. For ^13^C ramped cross-polarization magic angle spinning (CPMAS) spectra, the spinning rate used was 5 KHz and the temperature was set at 25 °C. FTIR spectra were measured on a Shimadzu FTIR IRAffinity-1 spectrometer (Shimadzu, Kyoto, Japan).

A hybrid quadrupole-time-of-flight (QTOF) mass spectrometer (MS) (Maxis Impact, Bruker Daltonics, Bremen, Germany) was utilized for the analysis and identification of the monomers. The QTOF system was equipped with an electrospray ionization interface (ESI), operating in positive ionization mode, with the following operation parameters: capillary voltage 6000 V; end plate offset −500 V; nebulizer pressure 1.0 bar; drying gas 1.0 L min^−1^; and gas temperature 150 °C. The QTOF MS system operated in full scan acquisition mode and recorded spectra over the *m*/*z* range 500–2200, with a scan rate of 1 Hz. External calibration of the mass spectrometer was performed with the manufacturer’s solution (sodium formate clusters), ensuring high mass accuracy. Stock solutions of 1 mg mL^−1^ were prepared for the monomers, by dissolving the appropriate amounts in acetone. Preliminary experiments for the optimization of MS (mainly source) parameters were conducted in order to enhance the ionization yield and obtain the highest sensitivity. Working solutions of 0.01 mg mL^−1^ were prepared and used for the infusion experiments. Infusion of the monomers’ solutions was performed under a constant flow of 180 μL min^−1^. Identification relied on the mass accuracy of the pseudomolecular ion of each monomer, as well as on the conformity of fit between the measured and the theoretical isotopic pattern. The potential presence of lower substitution degree monomers (with one or two branches) was examined and considered as an additional confirmatory criterion for the identification.

Scanning electron microscopy (SEM) was carried out on gold-coated dried aerogel filings, adhered on conductive double-sided adhesive carbon tape, using a Jeol JSM 5600 SEM instrument (JEOL Ltd., Tokyo, Japan). The system was operating at 20 kV, 0.5 nA, and 50 s time of analysis.

Thermogravimetric analysis (TGA) was conducted with a TA Instruments model TGA Q50 thermogravimetric analyzer (TA Instruments-Waters LLC, New Castle, DE, USA). Samples were placed in platinum crucibles. An empty platinum crucible was used as a reference. Samples were heated from ambient temperature to 800 °C in a 60 mL/min flow of N_2_ at a heating rate of 10 °C/min.

N_2_-sorption measurements were made on a Micromeritics Tristar II 3020 surface area and porosity analyzer (Micromeritics, Norcross, GA, USA). Skeletal densities were determined with He pycnometry, using a Micromeritics AccuPyc II 1340 pycnometer (Micromeritics, Norcross, GA, USA). Bulk densities (*ρ_b_*) of monolithic samples were calculated from their weight and natural dimensions.

Supercritical fluid (SCF) drying was carried out in an autoclave (E3100, Quorum Technologies, East Sussex, UK). Wet-gels were placed in the autoclave at 12 °C and covered with acetone. Liquid CO_2_ was allowed in the autoclave; acetone was drained out as it was being displaced by liquid CO_2_ (5×; 1 per 30 min). Afterwards, the temperature of the autoclave was raised to 45 °C and was maintained for 1 h. Finally, the pressure was gradually released, allowing SCF CO_2_ to escape as a gas, leaving dry-gels (aerogels).

### 2.1. Preparation of Poly(urethane acrylate) (aL-PUAc and aR-PUAc) Aerogels

#### 2.1.1. Synthesis of Dendritic Monomers

Acrylate-terminated dendritic monomers aL-Ac and aR-Ac were synthesized via reaction of two triisocyanates, Desmodur N3300 (1.0 g, 2.0 mmoL) or Desmodur RE (27% *w*/*w* solution of TIPM in ethyl acetate, 2.7 g, 2.0 mmoL), respectively, with PETA (1.8 g, 6.0 mmoL) in anhydrous acetone (24 mL), using DBTDL as catalyst (DBTDL/triisocyanate = 1:120 moL/moL). The reaction mixture was stirred at r.t. for 30 min under Ar. The solvent was then removed under vacuum. The crude product was redissolved in CH_2_Cl_2_, and hexane was added. Upon addition of hexane, aL-Ac formed a separate layer at the bottom of the flask. The top solvent layer was decanted, and the remaining viscous oil was dried under vacuum. aR-Ac was obtained as a precipitate that was collected and dried under vacuum. **aL-Ac**: Yield: 62%. ^1^H NMR (500 MHz, acetone-d^6^; Figure S1): δ (ppm) 6.39 (dd, 9H), 6.18 (m, 9H), 5.91 (dd, 9H), 4.55–4.0 (m, 24H), 3.83 (t, 6H), 3.10 (q, 6H), 1.7–1.1 (m, 24H).^13^C NMR (126 MHz, acetone-d^6^; Figure S2): δ (ppm) 165.3, 156.9, 149.2, 130.7, 128.2, 62.8, 60.2, 43.5, 42.3, 40.5, 27.6, 26.2. **aR-Ac**: Yield: 58%. ^1^H NMR (500 MHz, acetone-d^6^; Figure S3): δ (ppm) 8.60 (s, 3H), 7.49 (d, 6H), 7.07 (d, 6H), 6.5–6.0 (m, 27H), 5.47 (s, 1H), 4.5–4.0 (m, 24H). ^13^C NMR (126 MHz, acetone-d^6^; Figure S4): δ (ppm) 165.5, 154.5, 139.2, 137.9, 131.4, 129.9, 128.4, 118.6, 63.2, 51, 42.8. HRMS: calculated for C_64_H_67_N_3_O_24_H^+^ (three branches) *m*/*z*_th_ = 1262.4194, *m*/*z*_exp_ = 1262.4187 (Figure S5); calculated for C_50_H_49_N_3_O_17_H^+^ (two branches) *m*/*z*_th_ = 964.3141, *m*/*z*_exp_ = 964.3135 (Figure S6).

#### 2.1.2. Polymerization Reactions

Acrylate-terminated dendritic monomers aL-Ac and aR-Ac were polymerized using free radical chemistry. The quantity of anhydrous acetone was varied depending upon the desirable weight percent of the monomer in the sol. Gelation was induced by adding AIBN (AIBN/triisocyanate = 0.3:1 moL/moL) into the solution of the monomer in acetone. The resulting sol was stirred for 15 min at room temperature under Ar, then it was transferred to molds and left for gelation and aging for 24 h at 60 °C. The aged wet-gels were solvent exchanged with acetone (5 × 8 h), and finally dried to aerogels from supercritical fluid (SCF) CO_2_. Poly(urethane-acrylate) aerogels synthesized from aliphatic Desmodur N3300 or aromatic Desmodur RE are referred to as aL-PUAc-xx or aR-PUAc-xx, respectively, where xx denotes the percent weight of the monomer in the sol. All formulations are summarized in Table S1.

#### 2.1.3. Pyrolysis of aR-PUAc Aerogels

Poly(urethane acrylate) aerogels with an aromatic core (aR-PUAc) were transferred into an MTI GSL1600X-80 tube furnace (MTI Corporation, Richmond, CA, USA) (alumina 99.8% pure, 72/80 mm inner/outer diameters, 457 mm heating zone). The temperature was raised to 800 °C at 2.5 °C min^−1^ under flowing Ar (150 mL min^−1^) for 5 h. Afterwards, the flowing gas was switched back to Ar and the temperature was returned to room temperature at 2.5 °C min^−1^ under constant flow of Ar. All flow rates were set at 150 mL min^−1^. Yield: 20–30%.

## 3. Results and Discussion

### 3.1. Synthesis and Characterization of Dendritic Monomers aL-Ac and aR-Ac

**Scheme 1:**
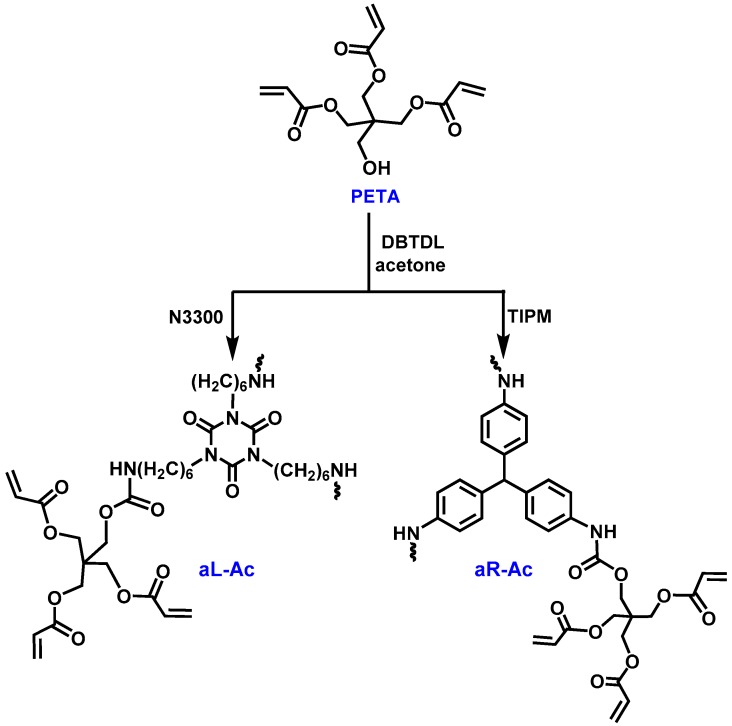
Synthesis of acrylate-terminated dendritic monomers aL-Ac and aR-Ac. PETA: pentaerythritol triacrylate; DBTDL: dibutyltindilaurate; TIPM: tris(4-isocyanatatophenyl)methane.

The synthesis of the acrylate-terminated dendritic monomers was carried out via reaction of one equivalent of triisocyanate (aliphatic N3300 or aromatic TIPM) with three equivalents of pentaerythritol triacrylate (PETA) (Scheme 1) at room temperature, and was catalyzed with an organometallic tin catalyst (dibutyltindilaurate; DBTDL), [44,45] in a 1:120 mol/mol ratio relative to the triisocyanate. The three starting materials are inexpensive chemicals of industrial interest that are produced in large quantities. The two monomers that were synthesized for this work bear either an aliphatic/flexible or aromatic/rigid core, and nine terminal functional acrylate groups (three acrylate groups for each of the three branches). As mentioned in the Introduction, previous work with similar star-shaped monomers involved the same aliphatic or aromatic core, but with only three terminal acrylate groups (one acrylate group at the tip of each of the three branches) [40].

The monomers were characterized with ^1^H and ^13^C NMR spectroscopy. aR-Ac was also characterized with high-resolution mass spectrometry (HRMS). Figures S1 and S3 show the ^1^H NMR spectra and Figures S2 and S4 show the ^13^C NMR spectra of aL-Ac and aR-Ac, respectively. The peaks observed in those NMR spectra were in agreement with the expected chemical composition of the monomers and with the peaks reported in the literature for similar monomers, bearing one acrylate group per branch [40]. More specifically, the most characteristic peaks were those in the olefinic region of the spectra, which confirmed formation of the acrylate monomers. Those peaks appeared at 6.39, 6.18, and 5.91 ppm (aL-Ac), or at 6.43, 6.37, and 6.20 ppm (aR-Ac) in the ^1^H NMR spectra, and at 130.7 and 128.2 ppm (aL-Ac), or at 131.4 and 128.4 ppm (aR-Ac) in the ^13^C NMR spectra. The ^13^C NMR spectra also provided information on the chemical composition of the monomers. In the spectrum of aL-Ac (Figure S2), the peaks corresponding to the carbonyls of the isocyanurate ring (C1) and the urethane groups (C8) are singlets, while the peak corresponding to the carbonyls of the acrylate groups (C12) is a doublet. Besides that, all other carbons of the acrylate groups (C9, C11–14) are also doublets. Similarly, in the spectrum of aR-Ac (Figure S4), the peaks corresponding to the urethane carbonyl (C6) and the carbons of the core (C1–C5) are singlets, while the peaks corresponding the carbons of of the acrylate groups (C9–12) are doublets. Those observations indicate that the starting material for the synthesis of the monomers (PETA) is a mixture of alcohols bearing two or three acrylate groups, with the alcohol that bears three acrylate groups being in excess. Both those alcohols react with the triisocyanates to yield monomers with three branches and six to nine acrylate groups. The fact that all three isocyanate groups have reacted is supported by the absence of the characteristic carbon peak at 120 ppm and by the fact that the C1 and C8 peaks (aL-Ac) are singlets. In addition, the HRMS spectrum of aR-Ac confirmed the presence of the three branches on the monomer (Figure S5). A peak assigned to a structure bearing two of the three branches was also detected (Figure S6) as a result of fragmentation during the measurement. Unfortunately, no HRMS data could be obtained for aL-Ac, as a result of fragmentation of the monomer during the measurement.

### 3.2. Synthesis of aL-PUAc and aR-PUAc Aerogels via Free Radical Polymerization

**Scheme 2:**
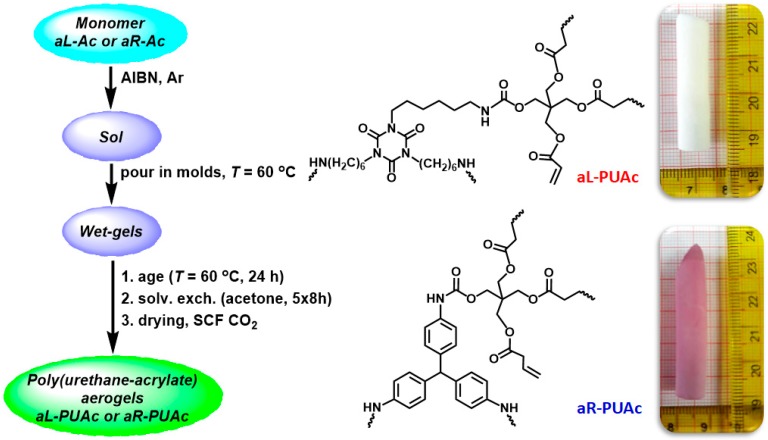
Synthesis of poly(urethane acrylate) aerogels aL-PUAc and aR-PUAc, chemical structures, and optical photographs. AIBN: 2,2′-azobis(isobutyronitrile).

Aerogels were synthesized from the two dendritic monomers of Section 2.1 above via free radical polymerization initiated by AIBN (Scheme 2). All wet-gels were aged for 24 h at 60 °C, were solvent-exchanged with acetone and dried with SCF CO_2_. An attempt was also made for ambient pressure drying from low surface tension pentane at 50 °C, but that process yielded materials with higher shrinkage, lower porosity, and lower BET surface areas, and thus it was not considered further. The polymerization of the monomers was quantitative as no free monomer could be detected by ^1^H NMR in the washings of the resulting wet-gels with acetone. The resulting materials are referred to as aL-PUAc-xx or Ar-PUAc-xx, where aL and aR refer to the aliphatic and the aromatic core, respectively, and xx indicates the % *w*/*w* monomer concentration in the sol. The monomer concentration was varied in the 1.5–12% *w*/*w* range. All aerogels were soft at low monomer concentrations and became more rigid as monomer concentration increased. In all cases, though, aerogels remained fragile even at high monomer concentrations. In spite of their lower density, the lack of flexibility is a notable difference between the aerogels presented here and the corresponding ones derived from trifunctional monomers reported in the literature [40]. Clearly, the difference is attributable to the higher degree of interparticle crosslinking, which in turn is traceable to the multifunctionality of the monomers. All formulations are summarized in Table S1. The chemical characterization and the properties of the new materials are described below.

### 3.3. Characterization of Aerogels aL-PUAc and aR-PUAc

**Figure 1:**
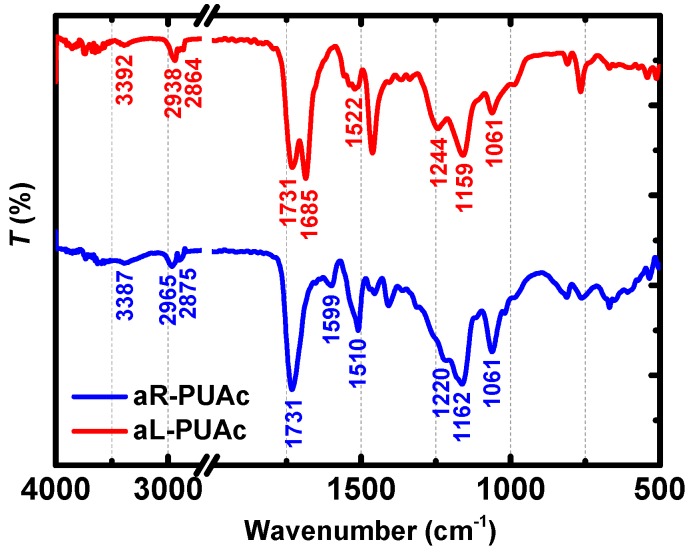
ATR-FTIR spectra of poly(urethane acrylate) aerogels aL-PUAc and aR-PUAc.

**Figure 2:**
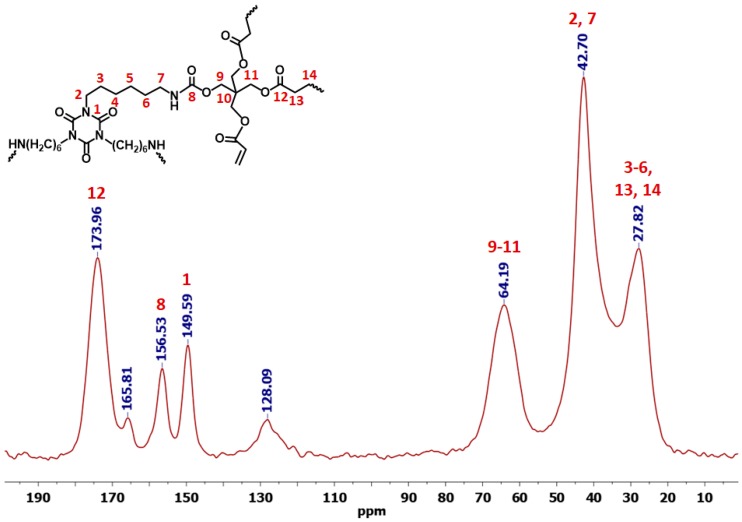

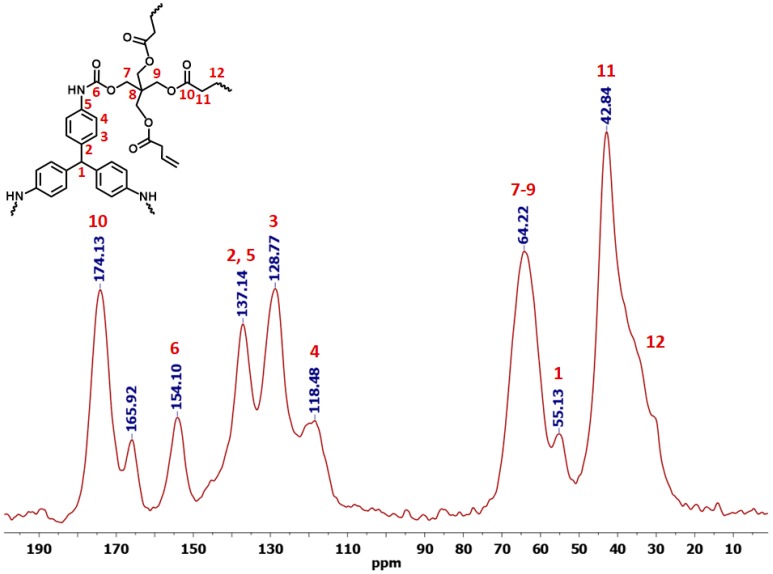
^13^C CPMAS NMR spectra of poly(urethane acrylate) aerogels aL-PUAc and aR-PUAc, as indicated.

Poly(urethane acrylate) aerogels aL-PUAc and aR-PUAc were characterized with ATR-FTIR and ^13^C CPMAS NMR, which are in agreement with the expected polymer structures and with the literature [40,41]. Representative spectra are shown in Figure 1 and Figure 2. The ATR-FTIR spectra of the aliphatic aL-PUAc and the aromatic aR-PUAc aerogels (Figure 1) have many common features: the stretching vibration of the urethane C=O at 1731 cm^−1^ for all aerogels, the stretching vibration of N–H without hydrogen bonding at 3392 (aL-PUAc) or 3387 (aR-PUAc) cm^−1^, the asymmetric stretching vibrations of aliphatic C–H at 2938 (aL-PUAc) or 2965 (aR-PUAc) cm^−1^, the symmetric stretching vibrations of aliphatic C–H at 2965 (aL-PUAc) or 2875 (aR-PUAc) cm^−1^, the bending vibration of N–H coupled to C–N stretching at 1522 (aL-PUAc) or 1510 (aR-PUAc) cm^−1^, the C–N stretching vibration at 1244 (aL-PUAc) or 1220 (aR-PUAc) cm^−1^, the asymmetric urethane C–O–C stretching at 1159 (aL-PUAc) or 1162 (aR-PUAc) cm^−1^, and the symmetric urethane C–O–C stretching at 1061 cm^−1^ for all aerogels. In addition, aliphatic aL-PUAc aerogels show the stretching vibration of the isocyanurate C=O at 1685 cm^−1^ and aromatic aR-PUAc aerogels show the stretching vibration of aromatic C–C at 1599 cm^−1^.

The ^13^C CPMAS NMR spectrum of aL-PUAc (Figure 2, top) shows three carbonyl peaks at 174 (C12), 156 (C8), and 150 (C1) ppm. The peaks at 166 and 128 ppm in the spectrum of the aerogels can be attributed to the carbonyl and the double bond of the monomer, respectively, showing that some of the acrylate double bonds remained intact during polymerization. Integration of the carbonyl peaks gave a ratio of C12 (including the peak at 166 ppm): C8/C1 equal to 1:0.26:0.29. The ^13^C CPMAS NMR spectrum of aR-PUAc (Figure 2, bottom) shows two carbonyl peaks at 174 (C10) and 154 (C6) ppm, with a ratio of 1:0.36 (by integration). As in the spectrum of aL-PAc, the presence of a peak at 166 ppm shows that some of the acrylate double bonds remained intact during polymerization. The ratios of the carbonyl carbons, as determined by integration, are consistent with the presence of six to nine acrylate groups per monomer.

**Figure 3:**
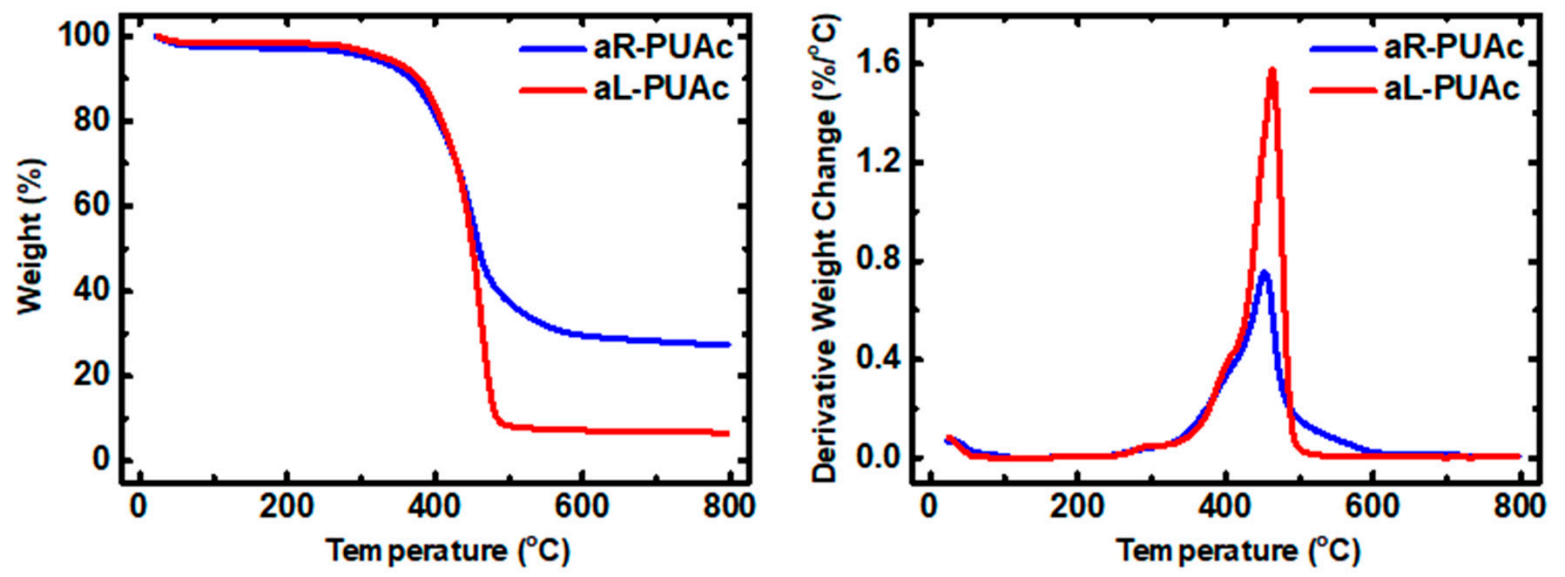
Weight loss with temperature (**Left**) and derivative weight loss with temperature (**Right**) forpoly(urethane acrylate) aerogels aL-PUAc and aR-PUAc, as indicated.

Thermogravimetric analysis (TGA) showed that the degradation process for all the above aerogels was very similar and was comprised of one major step (Figure 3, Left; Figure S7). The 1.9% and 3% weight loss of aL-PUAc and aR-PUAc aerogels, respectively, at 100 °C can be assigned to loss of residual solvent. The main degradation step happened in the temperature range of 300–450 °C (aL-PUAc) or 300–600 °C (aR-PUAc) and resulted in a residue of 6.7% and 27%, respectively. The higher residue of the aromatic versus aliphatic materials has been observed for other related materials in the literature [25,28], and is attributed to the almost complete thermal decomposition of the aliphatic branches of Desmodur N3300 on one hand [46], and the carbonization of the aromatic core from Desmodur RE (TIPM) on the other, which contains three aromatic rings separated by a single aliphatic carbon (a prerequisite for good-yield carbonization of aromatic polymers). Nevertheless, differential thermogravimetric analysis (DTG; Figure 3, Right; Figure S7) showed a main peak at 450–460 °C and a shoulder at about 400 °C for both types of aerogels, indicating that they share common initial degradation steps, which are attributed to the breakdown of the urethane (400 °C shoulder) and the acrylate moieties (main peak at 450–460 °C), respectively.

**Figure 4:**
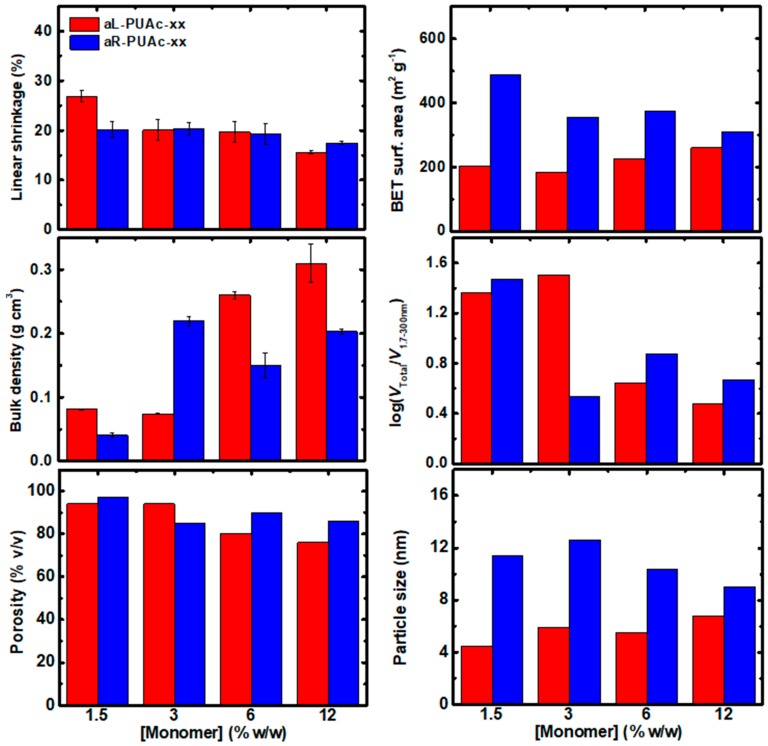
Comparison of relevant material properties of poly(urethane acrylate) aerogels aL-PUAc and aR-PUAc, as indicated.

**Figure 5:**
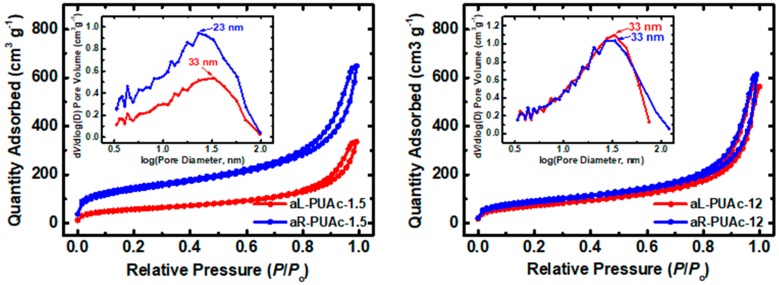
Representative N_2_-sorption isotherms for low-density (**Left**) and high-density (**Right**) poly(urethane acrylate) aerogels aL-PUAc and aR-PUAc, as indicated. Inserts show pore size distributions by the BJH method.

Material properties for all poly(urethane acrylate) aerogels are given in Table 1 and presented graphically in Figure 4. Representative N_2_-sorption isotherms for aL-PUAc and aR-PUAc are shown in Figure 5 together with pore size distributions as insets. N_2_ adsorption–desorption isotherms and pore size distributions of all materials are presented in the Supporting Information (Figure S8). In general, wet-gels showed no shrinkage during solvent exchanges, suggesting no major reorganization/swelling/de-swelling of the nanostructure during that process. On the other hand, all wet-gels shrank during SCF drying (linear shrinkage 16–20%), with the exception of wet-gels from the lowest-concentration aliphatic monomer (aL-PUAc-1.5), which exhibited a slightly higher shrinkage (27%). Skeletal densities were similar for all aL-PUAc and aR-PUAc aerogels (1.3 and 1.4 g cm^−3^, respectively), indicating no closed pores. As expected from the relative invariance of the skeletal densities and shrinkage with the concentration of the sols, higher concentration sols yielded aerogels with higher bulk densities and lower open porosities. The latter were always higher than 75%, reaching as high as 97% *v*/*v*. That was the case of aR-PUAc-1.5 at a bulk density of 0.041 g cm^−3^. By comparison, higher shrinkage noted with aL-PUAc-1.5 increased bulk density (0.081 g cm^−3^) and decreased porosity to 94% *v*/*v*. As all other things are equal, lower shrinkage, lower bulk density, and higher porosity in aR-PUAc-1.5 has to be attributed to the rigidity of their aromatic core.

In agreement with the shape of the N_2_-sorption isotherms (i.e., no saturation, narrow hysteresis loop; Figure 5), all aerogels gave *V*_Total_ >> *V*_1.7–300 nm_, indicating macroporous materials. The *V*_Total_/*V*_1.7–300 nm_ ratio was always ≥3 (reaching as high as 30) and decreased as the bulk density increased. This is consistent with skeletal frameworks formed by assemblies of secondary nanoparticles. Indeed, for the fraction of pores in the 1.7–300 nm range, the average pore diameter was always around 33 nm (by the BJH desorption method) in all materials, except for the low-concentration aromatic aerogels, for which the average pore diameter was a little smaller (23 nm). Average pore diameters were also calculated using the 4 *V*/*σ* method, whereas *V* was either the maximum volume of N_2_ adsorbed along the isotherm, or the volume (*V*_Total_) calculated from the bulk and the skeletal density of the materials (Table 1). In all aerogels, the average pore sizes obtained using *V*_Total_ were higher, and decreased with increasing bulk density.

BET surface areas for the aliphatic (aL-PUAc) samples had similar values at all densities (185–260 m^2^ g^−1^), and as a group, they were smaller than the BET surface areas for the aromatic (aR-PUAc) samples (311–488 m^2^ g^−1^), pointing to larger particles (see below). Significantly, a small fraction (4–8%) of the BET surface area of aromatic aerogels (aR-PUAc-xx, xx: 1.5–6) was assigned to micropores. This appears to be an intrinsic characteristic of aerogels based on the rigid aromatic core of TIPM, and has been observed with all polyuria [28,47], polyurethane [25,28,41,47], polyimide, and polyamide [48,49] aerogels.

**Figure 6:**
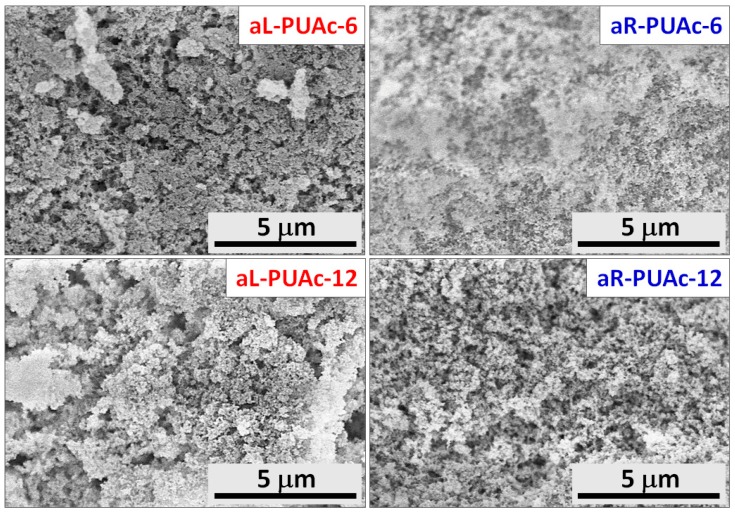
Representative scanning electron microscopy (SEM) images of poly(urethane acrylate) aerogels aL-PUAc and aR-PUAc, as indicated.

Representative SEM images are presented in Figure 6; SEM images for all samples are presented in Figure S7. Both aliphatic and aromatic aerogels at all concentrations show typical aerogel structures consisting of random assemblies of nanoparticles. Qualitatively, while the particle size is similar at all concentrations, it is evident that aL-PUAc aerogels consist of larger particles than aR-PUAc. Primary particle sizes (radii, *r*) were calculated from skeletal density (*ρ*_s_) and BET surface area data (*σ*) via *r* = 3/(*ρ*_s_ × *σ*). Data are presented in Table 1 and Figure 4. Primary particle sizes did not vary with the sol concentration and thus the density of the aerogels. However, the primary particle size of the aliphatic core aerogels (aL-PUAc) was about double the size of the aromatic core aerogels (aR-PUAc), which is attributed to the higher solubility expected from the more flexible (aL-PUAc) polymer than from the more rigid one (aR-PUAc).

If all data are considered together (porosity, relative pore volumes and pore sizes, BET surface area, and particle sizes), they point to a common nucleation mechanism for both aL-PUAc and aR-PUAc. Owing to the relatively low monomer concentration in all sols, polymerization proceeds slowly (all gelation times >3 h) and primary particles are formed when the growing polymer meets its solubility limit, which always happens at about the same size. Primary particles phase-separate and form secondary particles that ultimately aggregate and form the aerogel framework. Secondary particles were surface fractals. The fractal dimensions were calculated from N_2_-sorption data [50] and were very close to one another (2.54–2.59 for aL-PUAc, and 2.58–2.65 for aR-PUAc; Table 1). Fractal dimensions are characteristic physical properties of nanoparticle aggregates (in this case, secondary particles). Surface fractal secondary particles imply that their primary particles were closely packed, leaving a significant amount of mesoporosity between them. That mesoporous space included in secondary particles is transferred intact into the skeletal framework of the aerogel, thus explaining the decrease of *V*_Total_/*V*_1.7–300 nm_ ratio as the sol concentration, and thus the bulk density increases.

### 3.4. Pyrolysis of aR-PUAc Aerogels

**Figure 7:**
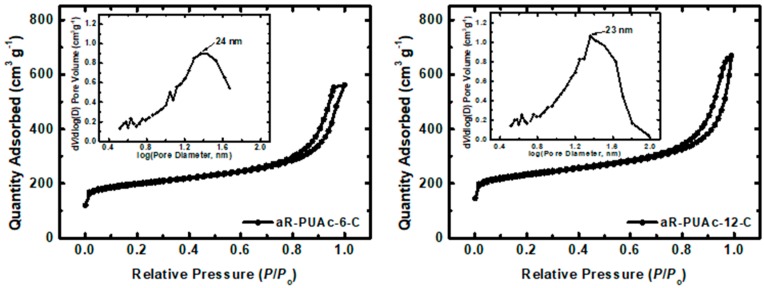
Representative N_2_-sorption diagrams for pyrolyzed aromatic poly(urethane acrylate) aerogels (aR-PUAc-xx-C), as indicated. Inserts show pore size distributions by the BJH method.

As mentioned above, aerogels with the aromatic core (aR-PUAc) left a significant residue during TGA (i.e., 27% at 800 °C/N_2_). Pyrolysis of those materials at 800 °C for 5 h under flowing Ar provided carbons at 20–30% yield. The N_2_-sorption isotherms are shown in Figure 7 and BET surface areas are reported in Table 2 for both parent and pyrolyzed aerogels for comparison purposes. The BET surface area of carbons derived from aR-PUAc-6 and aR-PUAc-12 was significantly higher than their parent aerogels (by ~2×), and the micropore surface area has increased significantly (to 56–58% of the total BET surface). Microporosity was probed with CO_2_ adsorption porosimetry up to 1 bar at 0 °C (Figures S9 and S10). Pore size distributions within the micropore range were calculated from the CO_2_ adsorption isotherms using the DFT method. Most of the micropores were distributed in the range of 0.4–0.8 nm. Micropore size distributions were very similar for all concentrations, and showed at least three maxima. Interestingly, after pyrolysis of aR-PUAc-1.5, which showed the highest BET surface area among the aromatic aerogels, the BET surface area decreased, while the micropore surface area increased. At the end, the BET and the micropore surface areas were about equal to one another, indicating a macroporous materials with microporous walls. For the aR-PUAc-6 sample, the amount of CO_2_ adsorbed was increased by 2.5× after pyrolysis.

### 3.5. Comparison of aL-PUAc and aR-PUAc Aerogels with Relevant Literature Materials

Although the aerogels of this work were chemically similar to the ones synthesized from the same trifunctional cores, but with only three acrylate groups per monomer [40], their properties were quite different. It is noted then that a direct comparison between the two groups of materials can be made only with samples prepared at a sol concentration of 12% (i.e., aL-PUAc-12 and aR-PUAc-12), as that was the only common concentration between the two studies. Specifically, as a direct result of the higher functionality of the materials of the present study (nine vs. just three acrylates at the tips of the three branches), the degree of crosslinking was higher and phase separation occurred earlier. Therefore, not only were particles smaller, but more importantly, they also had a higher number of dangling surface acrylates that crosslinked adjacent particles more strongly. That allowed gelation with sol concentrations in the range of 1.5–12%, while for aerogels from tri-acrylates monomers, the sol concentrations were in the range of 9–40%. Referring to aromatic aR-PUAc-12 aerogels relative to the literature materials [40], their BET surface areas were higher (311 vs. 139 m^2^ g^−1^) and their particle radii were smaller (6.8 vs. 17 nm). When it comes to the aliphatic aL-PUAc aerogels, differences become more pronounced: despite their higher bulk density (0.31 vs. 0.171 g cm^−3^) and lower porosity (76 vs. 86% *v*/*v*) compared with the literature materials [40], the aL-PUAc-12 aerogels of this study had remarkably higher BET surface areas than their tri-acrylate counterparts (260 vs. 2 m^2^ g^−1^) and much smaller particle radii (9 nm vs. 1.2 μm). It is the first time that a polyurethane aerogel based on an aliphatic triisocyanate has small nanosized primary particles; in all other cases we are aware of, including superelastic shape memory aerogels based on Desmodur N3300 [26,40,51], particles have been micron-sized, and most probably their growth mechanism was different.

In an effort to improve the properties of aerogels based on tri-acrylate star monomers, an attempt was made to rigidize the polymer by converting the three terminal acrylates to dangling norbornenes followed by gelation via ring-opening metathesis polymerization (ROMP) [40,43]. Albeit those aerogels based on tri-norbornene star monomers had improved properties compared with those based on their tri-acrylate analogues, for the same weight percent sols, aL-PUAc and aR-PUAc of the present study had lower densities, much smaller particle sizes, and much higher BET surface areas than the tri-norbornene star monomer aerogels. Motivated by the improved properties of aerogels from trinorbornene star monomers relative to those from triacrylate star monomers, we recently synthesized aerogels based on dendritic monomers with the same aromatic and aliphatic cores as in this study, but bearing nine terminal norbornene units [25]. Compared with those materials, acrylate-terminated monomers with an aromatic core (aR-PUAc) gave aerogels with higher bulk and lower skeletal densities (0.041–0.219 vs. 0.032–0.17 g cm^−3^ and 1.29–1.454 vs. 1.42–1.8 g cm^−3^, respectively), higher BET surface areas (311–488 vs. 188–294 m^2^ g^−1^), lower average pore diameters (8.2–13.4 vs. 11–123 nm), and smaller particle sizes (4.6–6.8 vs. 6.0–9.9 nm). Also, acrylate-terminated monomers with an aliphatic core (aL-PUAc) yielded wet-gels that shrank less during drying (16–27 vs. 27–34%), and the resulting aerogels had remarkably lower average pore diameters (10–13 nm vs. 13–88 nm) and smaller particle sizes (9.0–12.6 vs. 6.9–28.4 nm) than the corresponding norbornene-terminated ones.

## 4. Conclusions

In this work, new synthetic polymer aerogels based on dendritic-type urethane-acrylate monomers with aliphatic/flexible or aromatic/rigid cores have been synthesized. The monomers have nine terminal acrylate groups (three at the tip of each of the three branches), which were polymerized with AIBN via a free radical process. The resulting aerogels were softer at low monomer concentrations and more rigid at higher concentrations. They were characterized with ATR-FTIR and solid-state ^13^C CPMAS NMR. The porous network was probed with N_2_ sorption and SEM. The thermal stability of aerogels was studied with TGA. Aerogels were mostly macroporous materials with porosities >76% and a small percent of microporosity (4–8% for aromatic materials only), and high thermal stability (up to 300 °C). Materials with aromatic cores are carbonizable with satisfactory yields (20–30% *w*/*w*) to mostly microporous materials (56–58% of the total BET surface). Therefore, possible applications include use as electrode materials, and as adsorbents of CO_2_ and other pollutants.

The material properties of the aerogels synthesized in this work differ from those of analogous aerogels bearing only one acrylate moiety at the tip of each branch deriving from the same cores, mainly regarding their higher BET surface areas and smaller particle radii. Similar differences were also observed between the new materials and their analogues derived from norbornene-terminated monomers.

Finally, this study has confirmed that increasing the number of functional groups on the monomer increases crosslinking, and causes formation of aerogels with smaller particles and much larger BET surface areas. It is noteworthy that based on those principles, this seems to be the first time that a polyurethane aerogel based on an aliphatic triisocyanate has small nanosized primary particles. In all other aliphatic triisocyanate-based polyurethane aerogels we are aware of, particles have been micron-sized, and most probably their growth mechanism was different.

## Figures and Tables

**Table 1:** Material properties of poly(urethane acrylate) aerogels aL-PUAc and aR-PUAc.

Sample	Linear Shrinkage ^1^ (%)	Bulk Density *ρ_b_* (g cm^−3^)	Skeletal Density *ρ_s_* (g cm^−3^)	Porosity ^2^ *Π* (% *v*/*v*)	BET Surf. Area *σ* (m^2^ g^−1^) [Micropore Surf. Area] ^3^	*V*_Total_^4^ (*V*_1.7–300 nm_) ^5^ (cm^3^ g^−1^)	Av. Pore Diameter ^6^ (nm)	Particle Size ^7^ (nm)	Fractal Dimension ^8^ *D*_s_
aL-PUAc-1.5	27 ± 1	0.081 ± 0.001	1.29 ± 0.01	94	203	11.6 (0.5)	10.2 (228)	11.4	2.59
aL- PUAc-3	20 ± 2	0.074 ± 0.001	1.29 ± 0.02	94	185	12.7 (0.4)	10.2 (276)	12.6	2.58
aL-PUAc-6	20 ± 2	0.260 ± 0.006	1.275 ± 0.005	80	225	3.1 (0.7)	12.9 (55)	10.4	2.55
aL-PUAc-12	16 ± 2	0.31 ± 0.03	1.288 ± 0.001	76	260	2.4 (0.8)	13.4 (38)	9.0	2.54
aR-PUAc-1.5	20 ± 2	0.041 ± 0.003	1.40 ± 0.01	97	488 [38]	23.7 (0.8)	8.2 (193)	4.5	2.65
aR-PUAc-3	20 ± 1	0.219 ± 0.008	1.43 ± 0.02	85	356 [13]	3.9 (1.1)	13.4 (42)	5.9	2.60
aR-PUAc-6	19 ± 2	0.15 ± 0.02	1.454 ± 0.004	90	374 [18]	6.0 (0.8)	9.8 (64)	5.5	2.61
aR-PUAc-12	17.5 ± 0.3	0.203 ± 0.004	1.414 ± 0.006	86	311 [2]	4.2 (0.9)	12.2 (54)	6.8	2.58

^1^ Linear shrinkage calculated according to the following formula: (*y*_1_ + *y*_2_)/2, where *y*_1_ = 100 − [(*h*_fin_/*h*_st_) × 100] and *y*_2_ = 100 − [(*d*_fin_/*d*_st_) × 100; *h*: height, *d*: diameter. ^2^ Porosity calculated according to the following formula: (*ρ*_s_ − *ρ*_b_)/*ρ*_s_, where *ρ*_s_: skeletal density and *ρ*_b_: bulk density. ^3^ Micropore surface area via *t*-plot analysis, according to the Harkins and Jura model. ^4^ Total pore volume calculated according to the following formula: *V*_Total_ = 1/*ρ*_b_ − 1/*ρ*_s_. ^5^ Cumulative volume of pores between 1.7 and 300 nm from N_2_-sorption data and the BJH desorption method. ^6^ Calculated by the 4 *V*/*σ* method; *V* was set equal to the maximum volume of N_2_ adsorbed along the isotherm as *P*/*P*_o_ → 1.0. For the number in parentheses, *V* was set equal to *V*_Total_ from the previous column. ^7^ Particle radii calculated according to formula: *r* = 3/(*ρ*_s_ × *σ*). ^8^ Surface fractal dimension derived from N_2_-sorption data, calculated from the slope of the following plot: ln(*V*/*V*m) = (*D*_s_ − 3) ln(ln(*P*_o_/*P*)) + C, where *V* is the volume of N_2_ adsorbed at each equilibrium pressure *P*, *V*_m_ is the volume of N_2_ adsorbed in a monolayer, and *P*_o_ is the saturation pressure.

**Table 2:** Material properties of poly(urethane acrylate) aerogels with aromatic core before (aR-PUAc-xx) and after (aR-PUAc-xx-C) ^1^ pyrolysis.

Sample	Skeletal Density *ρ_s_* (g cm^−3^)	BET Surf. Area *σ* (m^2^ g^−1^) [Micropore Surf. Area] ^2^	Quantity of CO_2_ Adsorbed (mmoL g^−1^)
aR-PUAc-1.5	1.40 ± 0.01	488 [38]	0.63
aR-PUAc-1.5-C	2.0 ± 0.1	171 [154]	1.09
aR-PUAc-6	1.454 ± 0.004	374 [18]	0.97
aR-PUAc-6-C	2.15 ± 0.03	639 [360]	2.50
aR-PUAc-12	1.414 ± 0.006	311 [2]	0.84
aR-PUAc-12-C	2.3 ± 0.1	739 [429]	0.99

^1^ Pyrolyzed at 800 °C for 5 h under flowing Ar. ^2^ Micropore surface area via *t*-plot analysis, according to the Harkins and Jura model.

## Supplementary Materials

The following are available online at http://www.mdpi.com/1996-1944/11/11/2249/s1: Figure S1: ^1^H-NMR spectrum of aL-Ac in acetone-d^6^; Figure S2: ^13^C-NMR spectrum of aL-Ac in acetone-d^6^; Figure S3: ^1^H-NMR spectrum of aR-Ac in acetone-d^6^; Figure S4: ^13^C-NMR spectrum of aR-Ac in acetone-d^6^; Figure S5: Comparison between the theoretical (blue) and experimental (green) mass spectra of the pseudomolecular ion of aR-Ac. The species is identified according to the mass accuracy and the isotopic fitting information obtained; Figure S6: Comparison between the theoretical (blue) and experimental (green) mass spectra of the pseudomolecular ion corresponding to a fragment of aR-Ac bearing two of the three branches of the monomer. The species is identified according to the mass accuracy and the isotopic fitting information obtained; Figure S7: Weight loss with temperature (left) and derivative weight loss with temperature (right) for poly(urethane acrylate) aerogels aL-PUAc and aR-PUAc, as indicated; Figure S8: N_2_-sorption isotherms for poly(urethane acrylate) aerogels aL-PUAc and aR-PUAc, as indicated. Inserts show pore size distributions by the BJH method; Figure S9: Left: CO_2_ adsorption isotherms at 0 °C up to 1 bar for the aR-PUAc-xx aerogels, as indicated. Right: Pore size distribution calculated from CO_2_ adsorption data; Figure S10: Left: CO_2_ adsorption isotherms at 0 °C up to 1 bar for the pyrolyzed aerogels aR-PUAc-xx-C, as indicated. Right: Pore size distribution calculated from CO_2_ adsorption data; Table S1: Formulations for the synthesis of poly(urethane acrylate) aerogels aL-PUAc and aR-PUAc.
