# Supplementary material for: Poly(Urethane-Acrylate) Aerogels via Radical Polymerization of Dendritic Urethane-Acrylate Monomers
Source: Materials (Basel). 2018 Nov 12;11(11):2249. doi: 10.3390/ma11112249 (PMC6266260; doi:10.3390/ma11112249)
Supplement: Supplementary file 1 [file materials-11-02249-s001.zip › materials-380223-supplementary.docx]

POLY(URETHANE-ACRYLATE) AEROGELS VIA RADICAL POLYMERIZATION OF DENDRITIC URETHANE-ACRYLATE MONOMERS

Maria Papastergiou, Aspasia Kanellou, Despoina Chriti, Grigorios Raptopoulos, Patrina Paraskevopoulou*

Laboratory of Inorganic Chemistry, Department of Chemistry, National and Kapodistrian University of Athens, Panepistimiopolis Zografou, Athens 15771, Greece; [mapapast@chem.uoa.gr](mailto:mapapast@chem.uoa.gr) (M.P.); [aspasiakan@hotmail.com](mailto:aspasiakan@hotmail.com) (A.K.); [chritides@chem.uoa.gr](mailto:chritides@chem.uoa.gr) (D.C.); [grigorisrap@chem.uoa.gr](mailto:grigorisrap@chem.uoa.gr) (G.R.); [paraskevopoulou@chem.uoa.gr](mailto:paraskevopoulou@chem.uoa.gr) (P.P.)

***** Correspondence: paraskevopoulou@chem.uoa.gr (P.P.); Tel.: +30-210-727-4381; Fax: +30-210-727-4782

Received: date; Accepted: date; Published: date

**SUPPORTING INFORMATION**

**Table of contents**

|  |  | Page |
| --- | --- | --- |
| Table S1 | Formulations for the synthesis of poly(urethane acrylate) aerogels aL-PUAc and aR-PUAc. | S3 |
| Figure S1 | ^1^H-NMR spectrum of aL-Ac in acetone-d^6^. | S4 |
| Figure S2 | ^13^C-NMR spectrum of aL-Ac in acetone-d^6^. | S4 |
| Figure S3 | ^1^H-NMR spectrum of aR-Ac in acetone-d^6^. | S5 |
| Figure S4 | ^13^C-NMR spectrum of aR-Ac in acetone-d^6^. | S5 |
| Figure S5 | Comparison between the theoretical (blue) and experimental (green) mass spectra of the pseudomolecular ion of aR-Ac. The species is identified according to the mass accuracy and the isotopic fitting information obtained. | S6 |
| Figure S6 | Comparison between the theoretical (blue) and experimental (green) mass spectra of the pseudomolecular ion corresponding to a fragment of aR-Ac bearing two of the three branches of the monomer. The species is identified according to the mass accuracy and the isotopic fitting information obtained. | S6 |
| Figure S7 | Weight loss with temperature (left) and derivative weight loss with temperature (right) for poly(urethane acrylate) aerogels aL-PUAc and aR-PUAc, as indicated. | S7 |
| Figure S8 | N_2_-sorption isotherms for poly(urethane acrylate) aerogels aL-PUAc and aR-PUAc, as indicated. Inserts show pore size distributions by the BJH method. | S8 |
| Figure S9 | Left: CO_2_ adsorption isotherms at 0 °C up to 1 bar for the aR-PUAc-xx aerogels, as indicated Right: Pore size distribution calculated from CO_2_ adsorption data. | S9 |
| Figure S10 | Left: CO_2_ adsorption isotherms at 0 °C up to 1 bar for the pyrolyzed aerogels aR-PUAc-xx-C, as indicated Right: Pore size distribution calculated from CO_2_ adsorption data. | S10 |

**Table S1**. Formulations for the synthesis of poly(urethane acrylate) aerogels aL-PUAc and aR-PUAc.

| **Sample** | **Monomer** | | **Acetone** | | **AIBN** | |
| --- | --- | --- | --- | --- | --- | --- |
|  | **mass**  **(g)** | **mmol** | **mass**  **(g)** | **volume**  **(mL)** | **mass**  **(g)** | **mmol** |
| **aL-PUAc aerogels synthesized from aL-Ac dendritic monomer** | | | | | | |
| **aL-PUAc-1.5** | 0.73 | 0.52 | 47.9 | 60.5 | 0.098 | 0.60 |
| **aL-PUAc-3** | 1.74 | 1.24 | 55.3 | 69.8 | 0.098 | 0.60 |
| **aL-PUAc-6** | 1.22 | 0.87 | 19.1 | 24.2 | 0.099 | 0.60 |
| **aL-PUAc-12** | 1.22 | 0.87 | 8.9 | 11.3 | 0.099 | 0.60 |
| **aR-PUAc aerogels synthesized from aR-Ac dendritic monomer** | | | | | | |
| **aR-PUAc-1.5** | 0.89 | 0.70 | 58.0 | 73.0 | 0.098 | 0.60 |
| **aR-PUAc-3** | 0.46 | 0.36 | 15.3 | 19.3 | 0.098 | 0.60 |
| **aR-PUAc-6** | 1.47 | 1.16 | 23.1 | 29.3 | 0.099 | 0.60 |
| **aR-PUAc-12** | 1.47 | 1.16 | 10.8 | 13.6 | 0.099 | 0.60 |


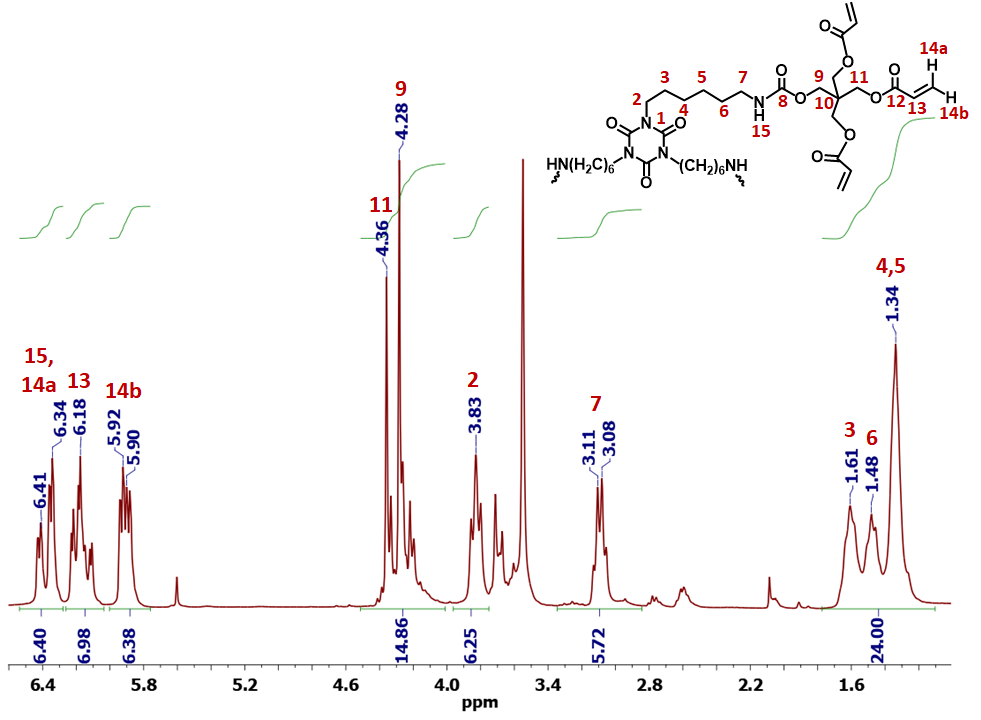


**Figure S1**. ^1^H-NMR spectrum of aL-Ac in acetone-d^6^.


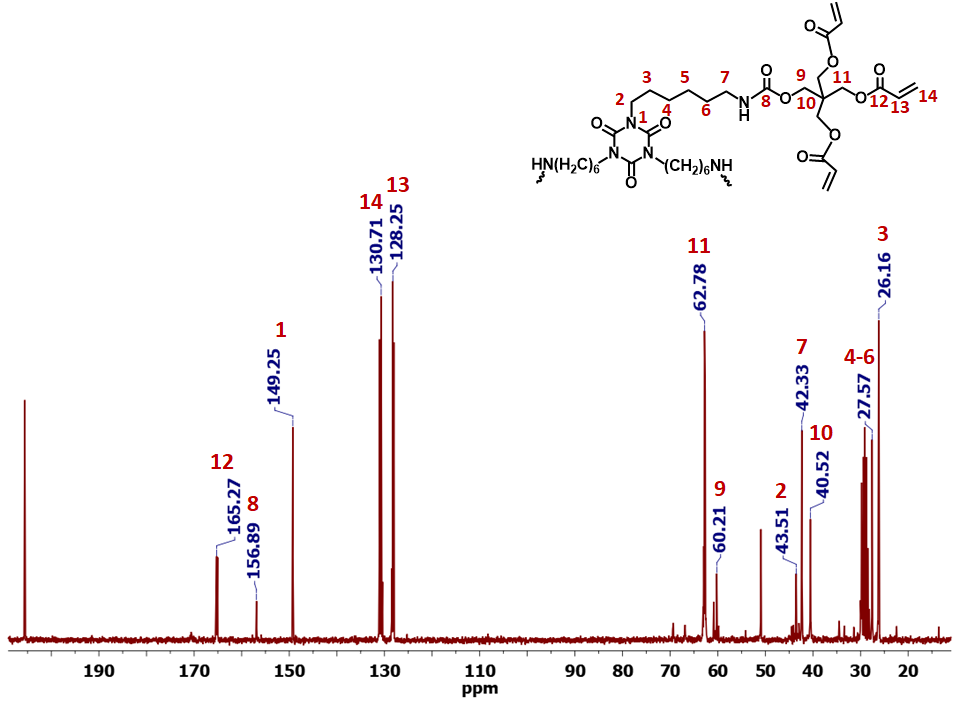


**Figure S2**. ^13^C-NMR spectrum of aL-Ac in acetone-d^6^.


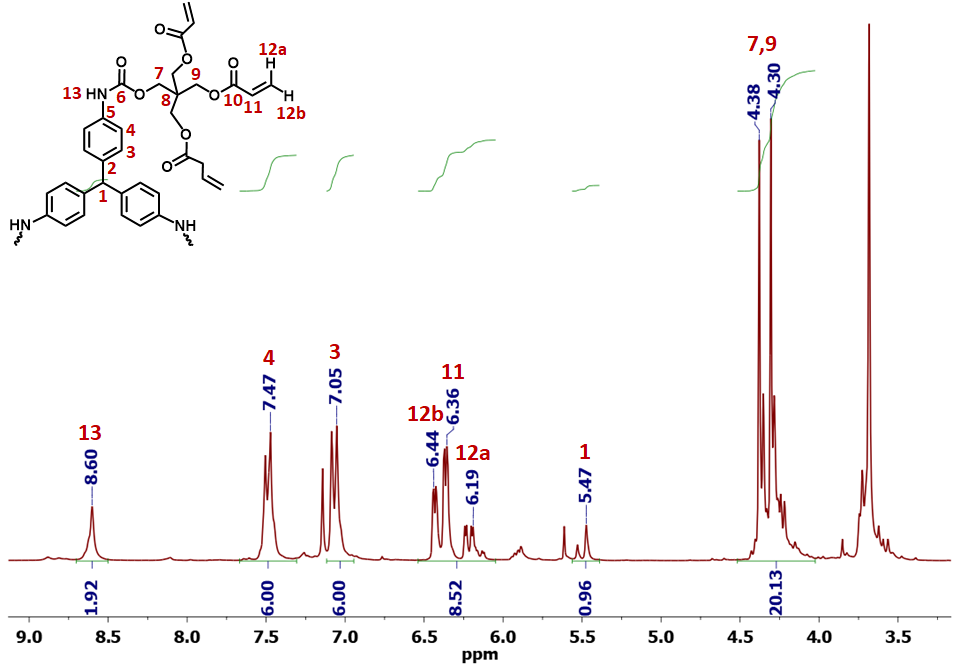


**Figure S3**. ^1^H-NMR spectrum of aR-Ac in acetone-d^6^.


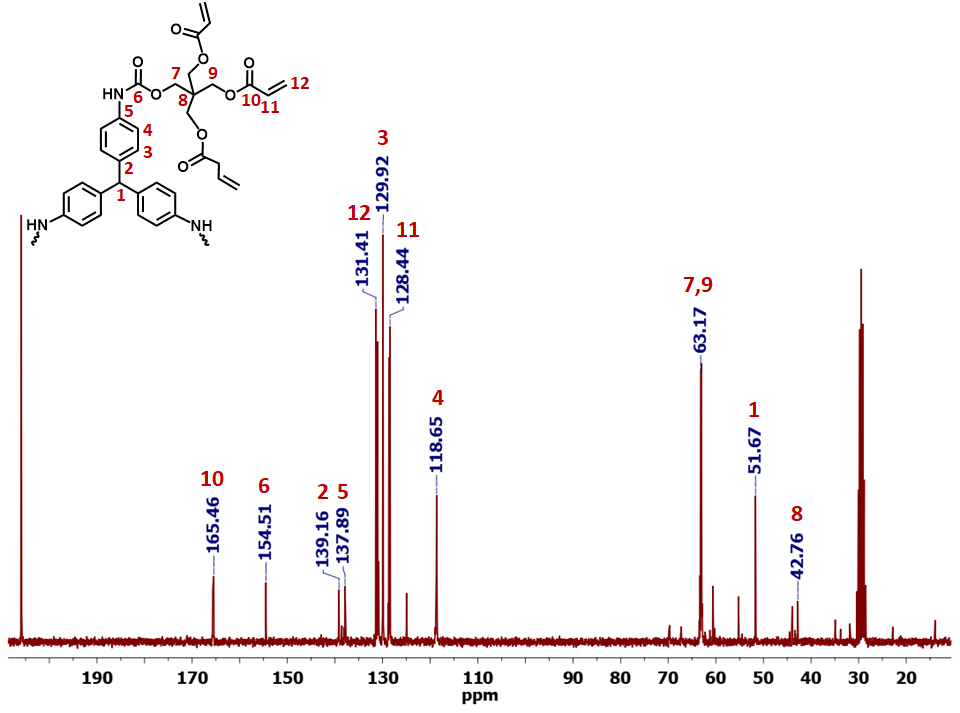


**Figure S4**. ^13^C-NMR spectrum of aR-Ac in acetone-d^6^.


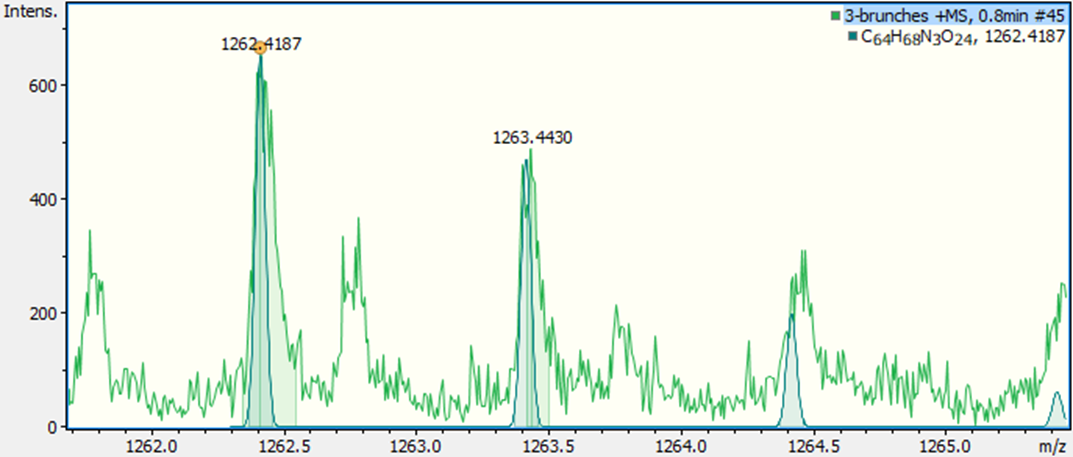


**Figure S5**. Comparison between the theoretical (blue) and experimental (green) mass spectra of the pseudomolecular ion of aR-Ac. The species is identified according to the mass accuracy and the isotopic fitting information obtained.


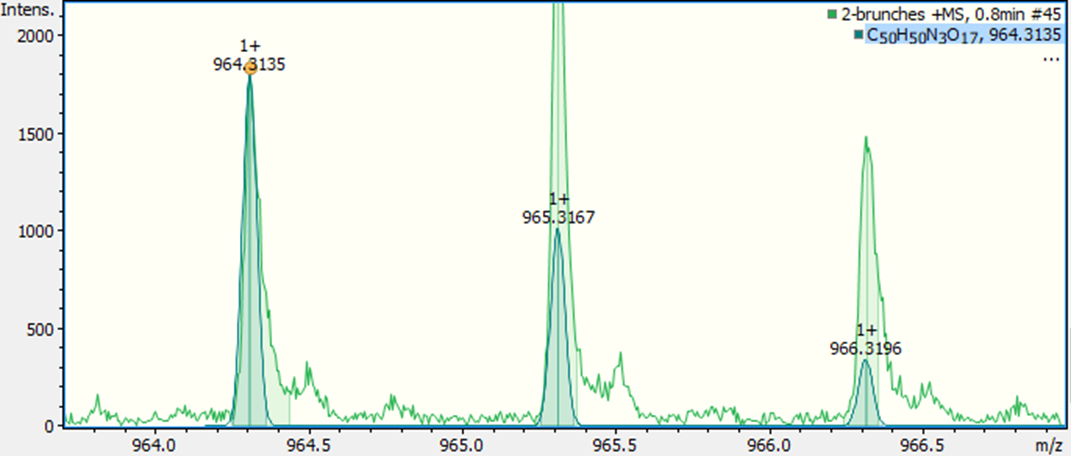


**Figure S6**. Comparison between the theoretical (blue) and experimental (green) mass spectra of the pseudomolecular ion corresponding to a fragment of aR-Ac bearing two of the three branches of the monomer. The species is identified according to the mass accuracy and the isotopic fitting information obtained.


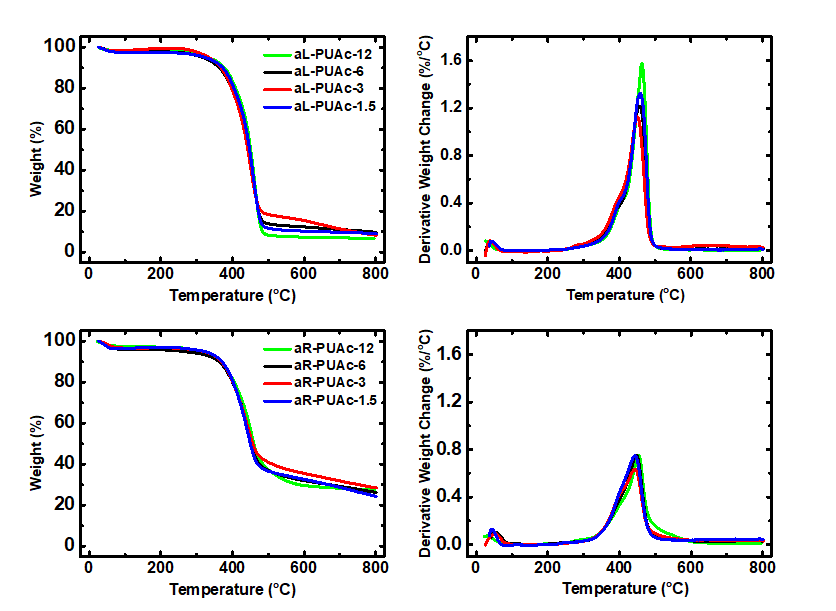


**Figure S7.** Weight loss with temperature (left) and derivative weight loss with temperature (right) for poly(urethane acrylate) aerogels aL-PUAc and aR-PUAc, as indicated.


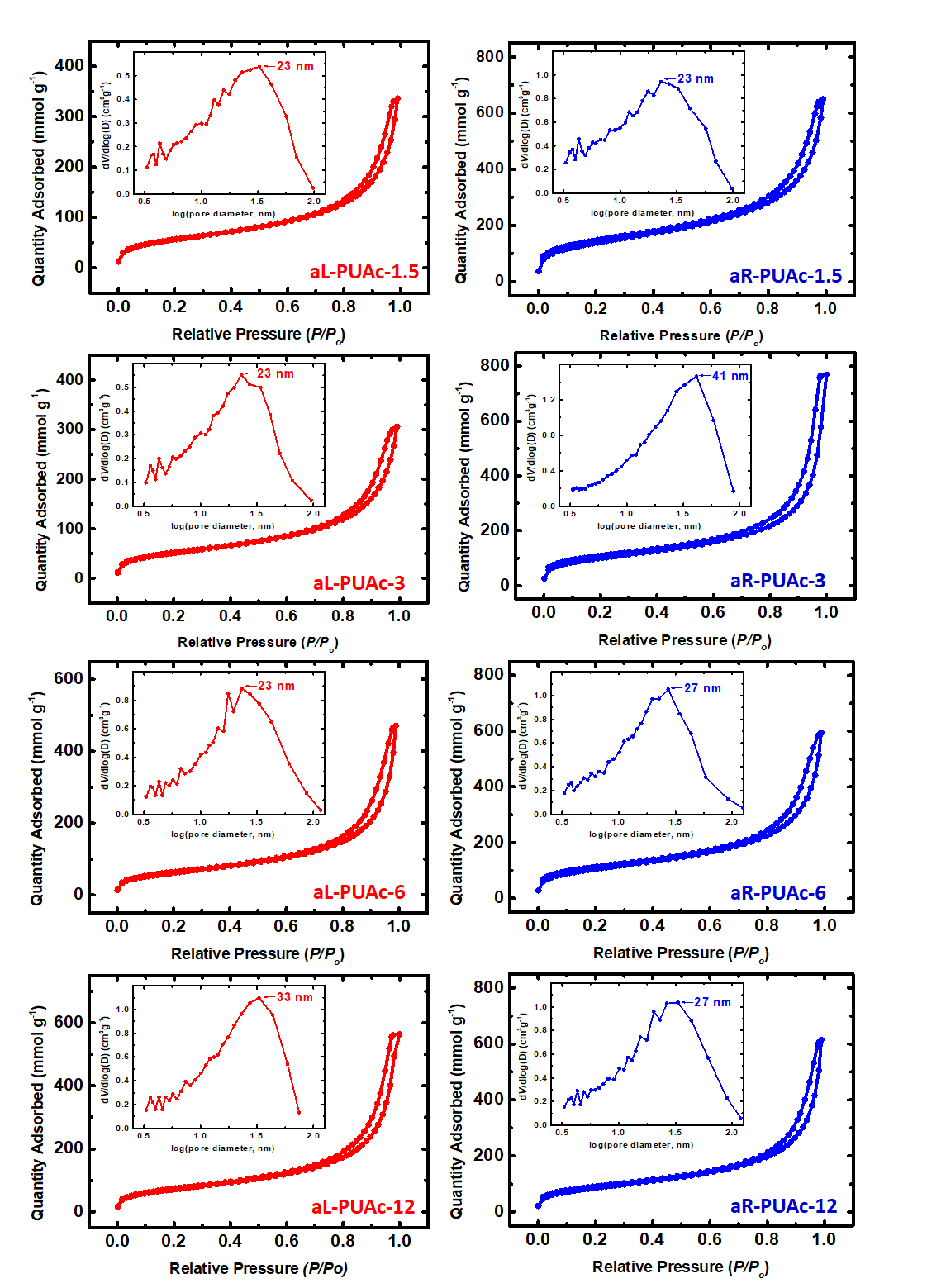


**Figure S8.** N_2_-sorption isotherms for poly(urethane acrylate) aerogels aL-PUAc and aR-PUAc, as indicated. Inserts show pore size distributions by the BJH method.


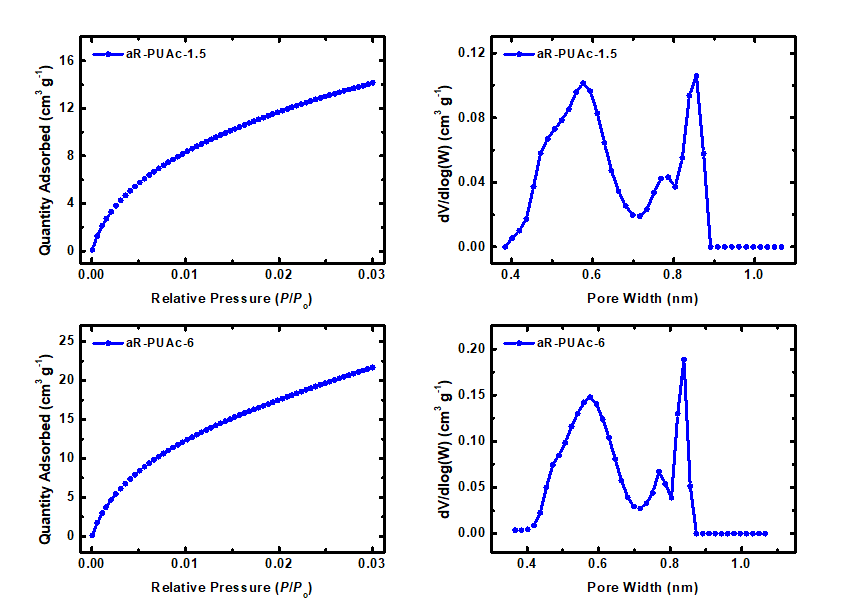


**Figure S9.** Left: CO_2_ adsorption isotherms at 0 °C up to 1 bar for the aR-PUAc-xx aerogels, as indicated Right: Pore size distribution calculated from CO_2_ adsorption data.


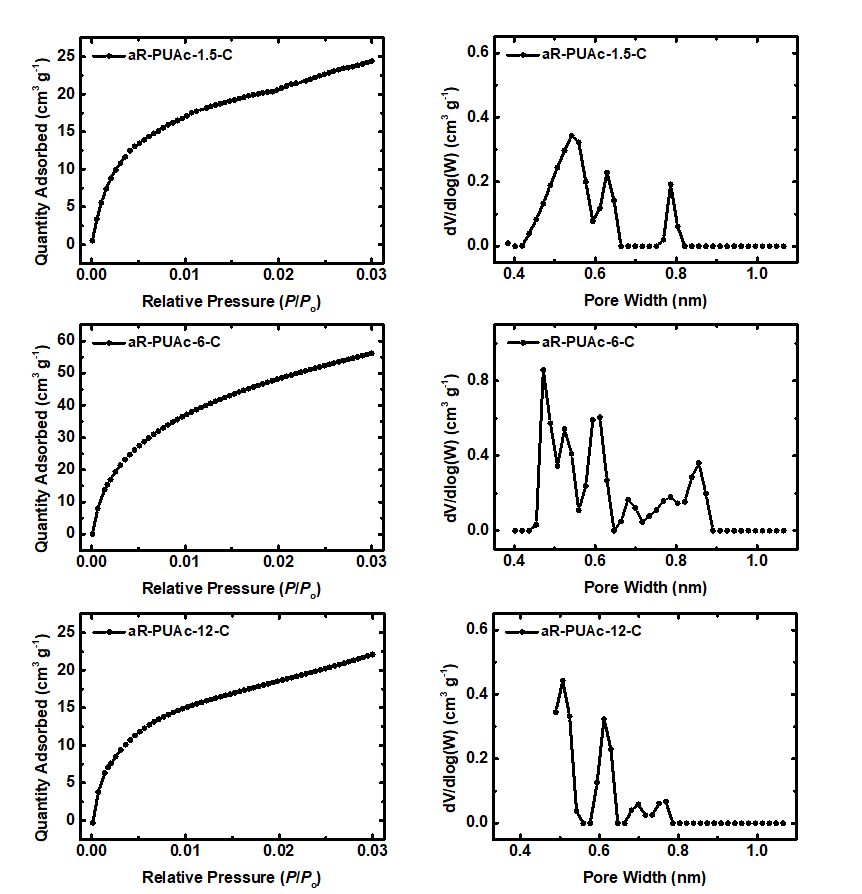


**Figure S10.** Left: CO_2_ adsorption isotherms at 0 °C up to 1 bar for the pyrolyzed aerogels aR-PUAc-xx-C, as indicated Right: Pore size distribution calculated from CO_2_ adsorption data.
